# Interorganisational Patient Safety Incident Reports: An Analytical Perspective

**DOI:** 10.1111/scs.70247

**Published:** 2026-04-24

**Authors:** Jaana Tuppurainen, Hannele Turunen, Kaisa Haatainen

**Affiliations:** ^1^ Department of Nursing Science, Faculty of Health Sciences University of Eastern Finland Kuopio Finland; ^2^ Department of Nursing Science, Faculty of Health Sciences University of Eastern Finland and Wellbeing Services County of North Savo Kuopio Finland

**Keywords:** care pathways, continuity of care, incident reporting, integrated care, patient harm, patient safety

## Abstract

**Aim:**

To obtain information on what kinds of patient safety incidents were reported between different healthcare organisations, which professional groups reported them, and what kind of development measures were planned to prevent recurrence of the patient safety incidents.

**Design:**

The data of this retrospective register study consisted of interorganisational patient safety incident reports (*n* = 1225) entered in the electronic incident reporting system from 2015 to 2021.

**Methods:**

The reports were sent to the university hospital from other healthcare organisations belonging to the university hospital's specific catchment area in Finland. Numeric data were analysed using descriptive statistical methods and open‐ended answers reporting development measures (*n* = 139) with inductive content analyses.

**Results:**

The majority of reported incidents between organisations were related to information flow and management (57%). The second most reported concerned medication, fluid therapies, blood transfusions, contrast, and marker patient safety incidents. Most of the reports were prepared by nurses, followed by physicians and other stakeholders. Many of the patient safety incidents reported were categorised as insignificant or low risk (67%). The type of incidents was usually ‘incidents occurring to the patients’, causing minor risk to the patient. Only a few interorganisational development measures were suggested.

**Conclusions:**

Collaboration between healthcare organisations should be improved to ensure timely and high‐quality information transfer regarding the safe continuity of patient care. Incidents should be regularly reviewed and the planning of preventive measures for recurrent incidents should be conducted by interorganisational multi‐professional teams, with the information in each organisation being disseminated at the unit level to improve safe care pathways.

**Implications for the Profession:**

To avoid variations in patient safety incident risk assessment and increase its reliability, the risk assessment should be performed by qualified assessors.

**Impact:**

This study showed that patient data transfer with adequate documentation and communication must be ensured for the continuity of care and patient safety. Development of interorganisational collaboration between professionals is needed to safeguard patient transfers from one healthcare organisation to another and ensure the patient's integrated care pathway.

**Patient or Public Contribution:**

No patient or public contribution.

## 1 Introduction and Background

Meeting the fundamental care needs of patients is the core of nursing and health services for ensuring quality and safe care. A series of patient safety incidents in the UK led to the development of a theoretical framework for nursing care, the Fundamentals of Care Framework (FOC), to explain, guide and potentially predict the quality and safety of care [1]. The FOC framework comprises three interconnected dimensions: establishing a trusting relationship between the care recipient and the care provider; integrating and addressing physical and psychosocial needs, alongside the care provider's relational actions in recognising and managing those needs; and creating a supportive context of care that enables relationship development and care integration [1, 2, 3]. Embedding this approach into everyday nursing practice demands significant organisational and interorganisational transformation and integration of care—for example, when a patient's care transfers from one healthcare unit or organisation to another.

Previous studies have shown that interorganisational collaboration in patient transfers needs to be improved [4, 5] to ensure patient safety in integrated care pathways. Goodwin [6] defines integrated care as an approach designed to overcome fragmentation in care, particularly when such fragmentation negatively affects patients' experiences and outcomes. Integrated care is important for people with complex medical conditions or long‐term care needs, and it involves a commitment to enhancing the quality and safety of services through continuous, collaborative, and co‐productive partnerships. Integrated care is a multidimensional and complex concept that can be supported by conceptual frameworks such as the renewed Development Model for Integrated Care (DMIC) [7]. The DMIC stresses care connectedness within a broader ecosystem and interorganisational collaboration. It comprises nine thematic clusters: client needs and networks; care delivery, coordination, and digital systems; performance management; proactive and preventive care; focused learning and reflection; interprofessional teamwork; roles and coordination; commitment to collaboration; and transparent entrepreneurship and governance. These clusters guide and evaluate integrated care development. Performance management is particularly relevant for patient safety, as it addresses outcome measurement, near‐miss and error analysis, feedback mechanisms, and improvement teams to strengthen collaboration and ensure safe patient transfers [7] In this paper, the FOC and DMIC models provide conceptual frameworks for discussing our results in the context of patient safety within integrated interorganisational care.

Integrated care uses interprofessional and interorganisational collaboration to ensure quality and safe care for patients [8]. However, collaboration can be hampered by different organisational structures and cultures, as well as by different professional identities [4]. These differences need to be considered when planning and implementing collaboration to deliver quality care and safe patient handovers in transition [4]. Collaboration in patient transfers is improved by interorganisational face‐to‐face meetings [9] and personal interaction [10], as well as individual actions by professionals [10, 11], such as additional verbal communication. This may have a positive impact on patient safety and the development of a patient safety culture when patients move from one care organisation to another [4, 9, 12]. Accurate structured documentation of patient information, timely transfer of that information, and adequate tailored recipient‐centred communication during patient transfers are associated with patient safety, continuity of care, and quality [13, 14, 15]. Delayed and inadequate data transfer during patient transfer from hospitals to primary care is still common [16], although it is suggested that the flexibility of the healthcare system helps ensure patient safety in interorganisational patient transfers [10]. Flexibility is reflected in temporary solutions and support measures developed by staff and patients during patient transfers and helps in the management of medication [10].

Electronic, voluntary incident reporting systems are used to monitor patient safety incidents (PSIs) in many healthcare organisations to improve patient safety by learning from mistakes and preventing them. For example, in Finland, hospital staff are encouraged to prepare reports, and the number of annual PSI reports has increased [17, 18]. However, our literature search demonstrated that there is a research gap in PSIs among healthcare organisations from a patient safety perspective. International research has shown that patient transfers between healthcare organisations do not work properly and are therefore not optimal for patient outcomes, highlighting the need for integrated care approaches to ensure continuity of care and patient‐centredness in interorganisational services [19, 20, 21]. This poses a risk to patient safety due to deficiencies and problems related to data transfer and inadequate documentation and communication of information [13, 20, 22, 23]. Thus, from the perspective of patient safety, there is a gap in the knowledge regarding integrated care pathways.

### 1.1 The Aim and Research Questions

The aim of the study was to produce information on patient safety incidents related to integrated care pathways that can be used to improve patient safety.

The research questions were as follows:

1. What kinds of interorganisational patient safety incidents were reported between different healthcare organisations?
2. Which professional groups reported patient safety incidents?
3. What kind of development measures were planned to prevent recurrence of the patient safety incidents?

## 2 Methods

### 2.1 The Study Context and Design

This study examined patient safety incidents within a Finnish university hospital district, where incident reports were submitted by healthcare organisations in the district, and the university hospital served as the recipient. In Finland, university hospitals are responsible for delivering highly specialised tertiary healthcare services within their designated regions. They maintain collaborative partnerships with secondary and primary healthcare providers in the area to ensure continuity and integration of care [24].

The design of this study was a retrospective register study consisting of reports of interorganisational patient safety incidents (PSIs), for which the electronic incident reporting system was established in 2015.

### 2.2 Data Description

The PSI reports (*n* = 1225) were from the electronic incident reporting system establishment for the 2015–2021 period, written by healthcare professionals. The notification recipient was the university hospital, and the PSI reporters were from 11 social and healthcare organisations and one support service unit located in the university hospital district.

These PSI reports provide structured quantitative numeric data on the location of the incident, nature of the incident, occurrence time, type of incident, risk category of the incident, consequences for the patient and treating unit, corrective actions, and reporter's professional group. The handler of the incidence report estimates the magnitude of risk based on the combination of the consequences of an event and the estimated probability of its recurrence. When assessing the magnitude of risk, generalisations need to be made by considering ‘average’ patients and ‘average’ situations, even though, in reality, patients are always individuals and healthcare situations vary. Nevertheless, based on the ‘average’, the risk is categorised as insignificant, low, moderate, significant or serious risk.

The PSI qualitative textual data were obtained from the open field of the PSI reports (*n* = 139), where the reporter or report handler freely provides additional textual information with regard to their view on how to prevent incident recurrence with a description of the measures to be taken [25].

### 2.3 Data Analysis

After obtaining permission from the hospital district to conduct the study and having received the data as an attachment via secure email from the hospital authorities, all data were initially pre‐processed by removing identifiers related to personal information. Subsequently, the numeric data set (*N* = 1225) was transferred to IBM SPSS Statistics 27 for analysis of the distribution of research variables in percentage terms and their interdependence using cross‐tabulation. The qualitative textual data relating to the views of the professionals with regard to the planning of the measures to be taken (*n* = 139) were analysed using inductive content analysis [26, 27, 28]. First, data were read several times to become familiar with the information. Second, phrases or expressions describing development measures that had been implemented or were planned for implementation were searched for in the text through reduction. Third, similar expressions were grouped, and they were compared for similarities and differences. Finally, the groups of expressions were named to describe the development measures to be taken in the interorganisational PSI reports.

### 2.4 Ethical Considerations

The study was conducted in accordance with the principles of good scientific practice [29]. Approval for the study (number 2494/2021) was obtained according to the procedures of the hospital district. The research material was stored in a data‐secure manner at all stages, and data protection was ensured according to the Finnish Data Protection Act (2018) [30]. The registry data of the incident reporting system were pseudonymised and could not be used to identify individual patients or those who had reported incidents. Healthcare units and organisations reporting PSI were classified and reported in such a way that no individual units or organisations could be identified.

## 3 Results

### 3.1 The Type of Reported Incidents

The number of interorganisational PSI reports (*N* = 1225) varied between 114 and 216 (9%–18%) per year in the seven‐year period (2015–2021). The location for reporting the PSI was usually a health centre (70%). The recipient university hospital units were most commonly acute service centres (40%) whereas the least common were mental health services (2%) (Table 1). The nature of the reported PSIs in care pathways was usually incidents involving patients (61%), near‐miss situations (38%), or other observation or development suggestions (1%). Typically, PSIs occurred on weekdays (73%) and took place in the morning or daytime (45%).

**TABLE 1:** PSI reporting health service organisation and the recipient university hospital unit (*N* = 1225, %).

	%
*PSI reporting health service organisation*	
Health centre	70
Home care or service housing unit	14
Hospital	13
Other health care unit (such as school healthcare, emergency medical care, student healthcare services, and health services administration)	3
*PSI recipient university hospital unit*	
Acute service centres	40
Operational centres	20
Core services centres	18
Medical services centre	9
Laboratory services	6
Mental health services	2
Other services (pathology unit, information management, secretary, patient transfer among others)	5

**FIGURE 1:**
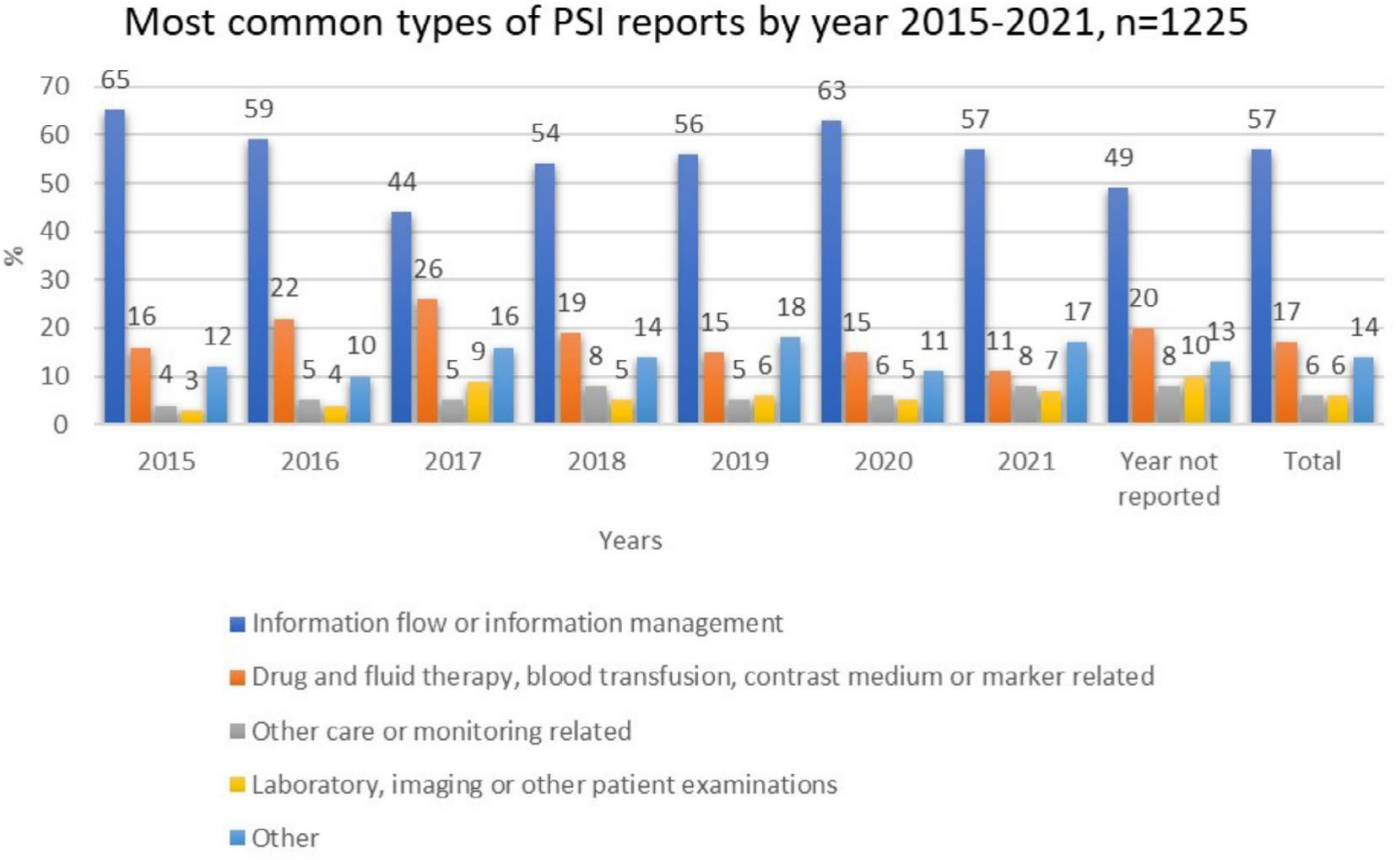
Most common types of patient safety incident (PSI) reports by year 2015–2021.

The most common type of the interorganisational PSI reports was related to information flow or management (57%) followed by drug and fluid therapy, blood transfusion, and contrast medium or marker (17%), whereas laboratory, imaging or other patient examinations (6%) were less reported PSIs (Figure 1). The information flow or management included incidents related to patient information management (documentation) events (25%), care or service arrangement (13%) and verbal information flow and communication (8%). Drug and fluid therapy, blood transfusion, contrast medium and marker PSIs typically included errors in recording (5%), prescription (3%), administration (2%), patient information management (documentation) events (1%), and supply (1%).

**FIGURE 2:**
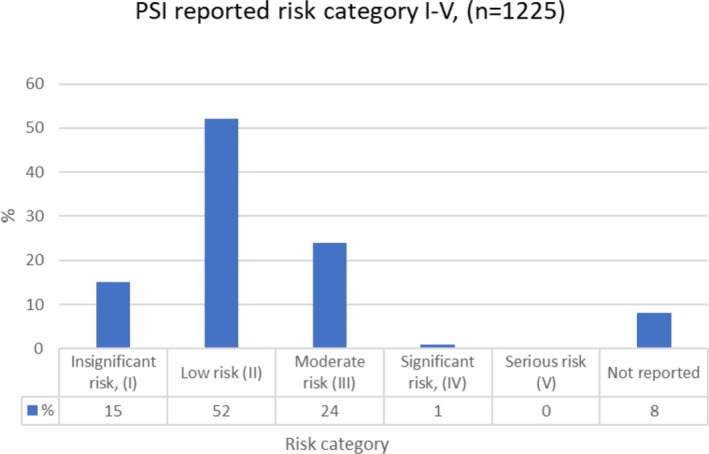
Risk categories of reported patient safety incidents (PSIs).

In two‐thirds of the cases, the risk category of the PSI reports was either insignificant (15%) or low (52%). No PSI reports were in the serious risk category. The risk categorisation was not reported in 8% of the reports (Figure 2). The levels of harm that affected patients were as follows: no harm (46%); mild (16%); moderate (5%); and serious (1%). The harm to the patient was unknown or unreported in 32% of the PSI reports. In terms of the consequences for the treating unit reported in the incident, these most often involved additional work or minor care and reputational damage (71%), while additional costs (4%) and prolonged care (3%) were reported less frequently. Typically, PSI corrective action was taken immediately in 66% of reported incidents in interorganisational settings; among these, immediate treatment, actions to prevent consequences and further harm, and patient observation or information accounted for 75%, 12% and 3%, respectively. The remaining reports did not have information regarding corrective actions.

### 3.2 Reporters of the Incidents

**FIGURE 3:**
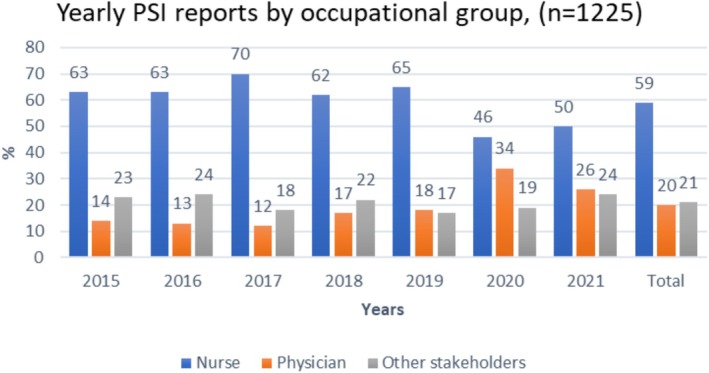
Yearly patient safety incident (PSI) reports by occupational group.

**FIGURE 4:**
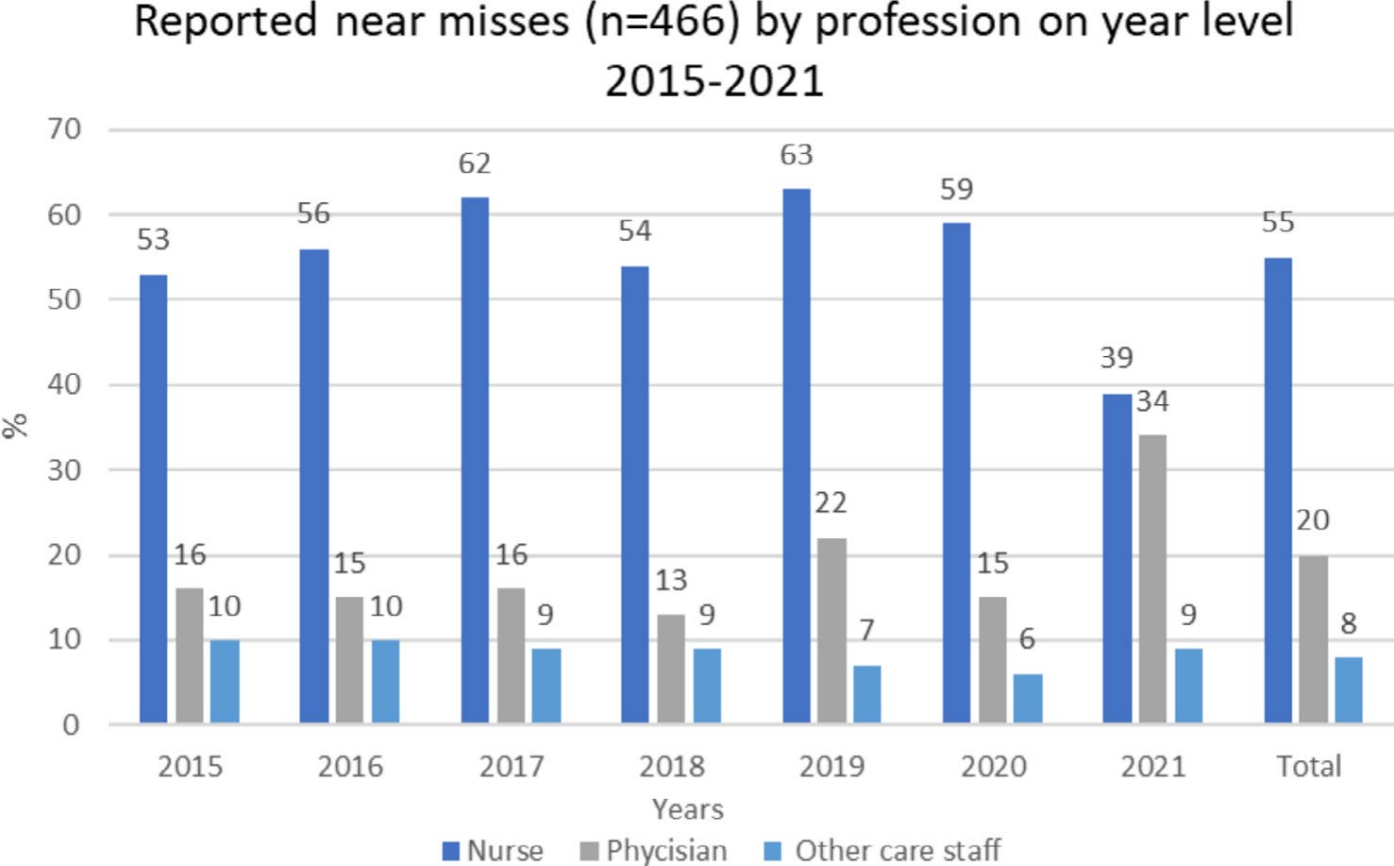
Reported near misses (*n* = 466) by profession on year level 2015–2021.

Interorganisational PSI reports were performed by registered nurses (59%), physicians (20%), and other stakeholders (21%), comprising various professionals such as practical nurses and other care staff. Registered nurses reported most of the ‘occurring to the patients by nature’ PSIs (62%) and the near misses (55%). The percentage of physicians reporting ‘occurring to the patients by nature’ PSIs was 21% and near misses 19%. For the period 2020–2021, the number of PSI reports made by physicians increased slightly, while the number of reports made by nurses decreased (Figure 3). In addition, for 2021, the study highlights an increase in near‐miss reporting by physicians (34%) and a parallel decrease in near‐miss reporting by nurses (39%) (Figure 4).

### 3.3 Development Measures to Prevent Recurrence of the Incidents

Of the analysed safety incident reports (*n* = 1225), the most common proposed preventive measure by far was providing information and discussing the event (75%, *n* = 924), but only in 11% (*n* = 139) of the incident reports was the developmental measure proposed by describing it in response to an open question. The development measures primarily described improving internal operations to ensure patient safety and were labelled as ‘Discussion, information, and agreeing the working practices’; ‘New policy implementation’; ‘Use of checklists, and Training and induction’.

#### 3.3.1 Discussion, Information, and Agreeing on Working Practices

The most common actions involved discussions and agreeing on working practices in various forums, such as ward meetings and multidisciplinary meetings, as well as increasing information sharing and reminders.

- ‘The matter was discussed and investigated regarding how the dictation and writing of medical records are carried out in urgent situations, and an agreement was made on the procedure for distributing approved texts as treatment feedback. The updating of patient medication lists was reviewed, and notation methods and archiving procedures in the medical record systems were agreed upon.’
- ‘Reviewed in a multidisciplinary manner. Distribution‐related practices to be reviewed. The doctor must remember to dictate the distribution information so that the feedback is sent.’
- ‘Information about the change in handling referrals has already been provided; it will take a transitional period for the change in question to be implemented.’

#### 3.3.2 New Policy Implementation

New policies were developed or updated, particularly for safety incidents related to medication management, laboratory processes, and multidisciplinary cooperation.

- ‘Planning for a “medication licence” has begun (medication management responsibility group, internal medicine specialist, and ward pharmacist).’

#### 3.3.3 Introduction of Checklists

The use of checklists to ensure patient safety was highlighted, especially in connection with patient discharge and follow‐up care.

- ‘Doctors' checklist created. RN (Registered Nurse) resources increased. Discharge model to be implemented from Monday.’
- ‘The matter has been discussed, and the discharge checklist should correct this. The checklist will be used for every transferring/discharged patient from now on.’

#### 3.3.4 Training and Induction

Training and induction were used to support the implementation of new operating models, such as the Identify, Situation, Background, Assessment, Recommendation (ISBAR) structured communication tool to strengthen staff competence to prevent the recurrence of safety incidents.

- ‘Almost everyone has received ISBAR training, but the knowledge from the training has not yet translated into practical action. ISBAR reporting guidelines to be made more visible (laminated instructions in the office) and reporting to be practised in practical situations.’
- ‘Inadequate reporting, failure to update the medication list, and missing final assessments are events that repeatedly compromise patient safety. A systematic, uniform induction is being planned for doctors… A theme week on reporting will be organised for the nursing staff…’

## 4 Discussion

The key findings of this study were that most of the reported PSIs between healthcare organisations were related to information flow and management, most of which were reported by nurses, with physicians being second in reporting them. Furthermore, PSI reporting led to limited interorganisational development measures. Previous studies have shown that patient transfers between healthcare organisations do not function properly; therefore, they are not optimal for positive patient outcomes. This poses a risk to patient safety due to deficiencies and problems related to data transfer, inadequate documentation, and communication of information [13, 20, 22, 23]. The different electronic information systems and interfaces used by healthcare organisations, as well as usability and functionality problems, make it difficult to transfer patient data, increase the possibility of errors, and jeopardise the continuity of patient care between healthcare organisations [23]. In addition to the development of information technology solutions to ensure continuity of care, patient safety in care pathways must be developed through collaboration between organisations. Different organisational structures and cultures, as well as different professional identities that hinder collaboration, need to be considered when planning and implementing the development of collaboration for quality care and safe patient transfer [4]. To systematically improve interorganisational integrated patient care, the DMIC model can serve as a valuable framework for managing the multidimensional and complex nature of healthcare. It emphasises care connectedness and provides structure for improvement activities, such as measuring and analysing outcomes, assessing the value of care chains and networks, conducting near‐miss and error analyses, implementing feedback mechanisms, and supporting improvement teams. These elements collectively help enhance and manage performance levels within integrated interorganisational collaboration [7].

According to this study, nurses and physicians reported the highest number of interorganisational PSIs. Seven years of data (2015–2021) show that typically reported PSIs were of an ‘occurred to a patient’ nature. While a substantial proportion of PSIs were categorised as insignificant or low‐risk and no serious risk incidents were reported, 8% of the reports lacked any risk assessment. Furthermore, in one third of the PSIs the level of harm to the patient was unknown or unreported. Such incidents typically result in additional work for staff, minor treatment, and/or reputational damage to the hospital [31]. These results challenge healthcare staff to be more responsible and competent in reporting interorganisational PSIs so that they can be used effectively to ensure and improve patient safety in integrated care [1, 2, 3].

Although no PSI reports were classified as serious risks, this does not necessarily indicate underreporting of serious PSIs. In Finland, reporting severe PSIs is statutory [32] and requires healthcare professionals to notify the person in charge of the service unit without delay. Because of this, serious PSIs may not appear in electronic, voluntary incident reporting systems [25]. To avoid risk misclassification, more attention should be paid to the assessment of PSI risk, and such assessments should be conducted by independent, skilled, and qualified assessors. In Finland, the patient safety risk assessment of individual incident reports is performed independently by the line manager of each healthcare unit receiving the report, which may lead to variations in risk‐level assessments and thereby reduce reliability [18]. This consideration is critical, as previous well‐documented adverse events in patient safety underscore the importance of maintaining patients' fundamental care needs and fostering a trusting relationship between patients and healthcare professionals. These principles should remain central at all levels—micro (individual), meso (ward or organisational) and macro (interorganisational and policy) – as articulated in the Fundamentals of Care (FOC) framework, which provides guidance for delivering person‐centred fundamental care [1, 3, 33]. Furthermore, the reputational damage within a healthcare organisation may significantly contribute to workforce shortages. Skilled and professionally committed healthcare practitioners are often reluctant to seek employment in institutions perceived as having a poor reputation, which can undermine recruitment and retention efforts [1].

In this study, interorganisational incident reports led to limited interorganisational development measures. Typically, development measures led to internal development activities within university hospitals to prevent recurrence and ensure continuity of care. These measures focused on providing internal information, discussing what happened, increasing information sharing, and reminding people of the course of action. To some extent, the new internal checklist had been used as a development measure in the context of patient discharge or follow‐up care. The data used in this study did not provide information on how the organisation learned from the development of measures to prevent the recurrence of a PSI. The organisation receiving the PSI report should pay close attention and follow up on similar recurring incidents to prevent recurrence and verify the effectiveness of the development measures to ensure sustainable patient safety in interorganisational care pathways.

## 5 Study Limitations

Due to the unvalidated nature of the incident reporting system's data, the report represents only the reporter's own view and assessment of the incident, which may have influenced the reliability of the data. The quantitative data set was extensive, covering incidents from 11 healthcare organisations within the university hospital district. These data describe patient safety issues that occurred in the region's hospitals and thus provide valuable insights for interorganisational learning and for improving operations along integrated care pathways. Variations in how the reporter had described the development measures taken in the open question may have influenced the researcher's interpretation of the qualitative data. Additionally, the qualitative analysis of the data for developmental measures only covered a subset of the incident reports (*n* = 139). Therefore, it is possible that a more detailed qualitative analysis of the entire data set would find additional descriptions of developmental measures. However, in the inductive content analyses saturation was achieved, when the content of the data began to repeat itself, thus the data and the results can be considered trustworthy [34]. The study did not examine the patients' perceptions of the reported PSIs. Despite the aforementioned limitations, the study was carefully conducted to avoid misinterpretation and bias.

## 6 Conclusions

This study showed that patient data transfer with adequate documentation and communication must be ensured for the continuity of patient care in interorganisational collaboration. Further development of interorganisational collaboration between professionals is needed to safeguard patient transfers from one healthcare organisation to another and to ensure the patient's integrated care pathway. Interorganisational patient safety incidents should be regularly reviewed and analysed by interorganisational multiprofessional teams, and the information in each organisation should be disseminated at the unit level to improve care pathways and patient safety. The risk assessment process for healthcare professionals reporting PSIs should be performed by independent, skilled and qualified assessors to enable a coherent and reliable risk‐level assessment. More focus should be placed on monitoring recurring PSIs between healthcare organisations to ensure the effectiveness of the development measures implemented and to prevent PSI recurrence.

## Author Contributions

All authors have agreed on the final version to be published and meet the following criteria (recommended by the International Committee of Medical Journal Editors): Jaana Tuppurainen (JT), substantial contributions to conception and design, analysis and interpretation of data, drafting the manuscript, and revising it critically for important intellectual content. Hannele Turunen (HT), substantial contributions to conception and design, analysis and interpretation of data, drafting the manuscript, revising it critically for important intellectual content. Kaisa Haatainen (KH), substantial contributions to conception and design, analysis and interpretation of data, revising it critically for important intellectual content.

## Funding

The authors have nothing to report.

## Disclosure

Statistics: The statistics were checked prior to submission by Biostatistician, MSc, Tuomas Selander, tuomas.selander@pshyvinvointialue.fi.

## Conflicts of Interest

The authors declare no conflicts of interest.

## Data Availability

The data that support the findings of this study are available from the corresponding author upon reasonable request.
